# Keystone Veterinary Medical Association

**Published:** 1897-01

**Authors:** W. L. Rhoads

**Affiliations:** Secretary


### KEYSTONE VETERINARY MEDICAL ASSOCIATION.

The Association was called to order for the November meeting on the 10th instant, with the following members of the profession in attendance: Thomas B. Rayner, H. J. McClellan, O. G. Noack, S. H. Johnston, H. A. Christman, F. S. Allen, H. P. Eves, Charles T. Goentner, John R. Hart, W. Horace Hoskins, Charles Lintz, James T. McAnulty, L. S. Pearson, James B. Rayner, W. L. Rhoads, and a number of veterinary students from the Veterinary Department of the University of Pennsylvania.

The election of officers, having been postponed from the October meeting, was taken up at this time and resulted in the election of Dr. John R. Hart, President; James B. Rayner, Vice-President; Francis S. Bridge, Treasurer; W. L. Rhoads, Secretary; with the following Board of Trustees: Charles T. Goentner, F. S. Allen, H. P. Eves, W. H. Hoskins, and L. Pearson.

Dr. Hoskins suggested that as Dr. E. A. A. Grange was one of the originators of the Keystone Veterinary Medical Association, he be given one of the membership certificates. Dr. Goentner raised the objection that under such ruling anyone who had been a member could procure a certificate by having a present member champion his cause; he suggested that, in consideration of the work Grange had done for the Association and the great amount of good work he was doing for the profession in his present position, we make him an honorary member. This matter was left for further discussion at the December meeting.

Dr. Benjamin Lee, Secretary of the State Board of Health, now addressed the Association on "Legislation of Mutual Interest to the People, as represented by the State Board and the Veterinarians as Sanitarians." During this talk he read from the proceedings of the U. S. V. M. A. a number of resolutions and gave the veterinarians great credit for a number of things that were done, and said in a number of ways they were already ahead of the medical men. He then explained how the veterinarians might cooperate with the State Board. He considered our laws as being inadequate in regard to the inspection of our meat- and milk-supply, saying that all tuberculous meat should be stamped as such, and trichinous pork, though it may be sterilized by cooking, is certainly not nice, and he believes the fat only should be used. The inspection of milk, to be good, should begin at the dairy and should be made by competent veterinarians, as no physician is as capable as the veterinarian of judging from beginning to end. This inspection, with an insistance of the tuberculin test, is the only safeguard for the people, and it is to be hoped that soon legislation on this point will be taken up by the State instead of being just municipal. He spoke of the wonderful decrease in the death-rate of Buffalo within the last five years, since the water could not be mistaken for ink nor pea-soup; the streets were good, the milk-supply well guarded, and the health officer a competent physician; the death-rate has decreased to such an extent that the actual number of deaths last year (with the great increase of population) was less than it was five years ago. He spoke of the number of diseases common to the lower animals and man, and said it would be interesting to know to what an extent the domestic animals are carriers of disease, though not themselves affected. Whooping-cough has been fatal to the dog; diphtheria quite common to the dog and cat; they have the range of the house, and their fur is an admirable carrier of these as of sundry other disease-germs. He hopes the people will soon awake to the necessity of a health officer in every township; this would in a great measure assist in keeping complete vital statistics; he claims it is an absolute disgrace not to have this registration compulsory throughout the State. The State Board fund is inadequate, being but $6000, $2000 of which is used as salary for the secretary and $2000 for clerks, leaving but $2000 for the investigation of diseases, watching streams, and other imperative duties.

Dr. Pearson said the inspection of meat, milk, and dairies was done in foreign countries with great thoroughness, and claims in the inspection of milk the important point is the inspection of the cows and their surroundnings, as the specific gravity and color may be all right, yet the cattle may have tuberculosis; water may contain typhoid germs; attendants may have diphtheria or some kindred disease. Hoskins excused the State Board for sending one of their members to examine the piggeries which caused so much trouble, but felt they did it on account of the inadequate funds. Noack said that all cities of the third class could employ a food commissioner, and he might be a veterinarian; he then explained why he had troubles of his own with the people of Reading and vicinity. Eves said in Delaware the veterinarian did not have enough pull, as their bill framed on contagious diseases was never heard of from the committee-room. Wilmington has a farmer for milk-inspector. Allen spoke of New York having a law compelling both owner and veterinarian to report and destroy all animals suffering from contagious diseases. Just before adjournment Dr. Hoskins, in behalf of his co-workers, with one of his silvery speeches, presented Secretary Rhoads with a magnificent lamp as a mark of their approbation of his recent venture. This (the presentation) being a complete surprise to the Secretary, was but feebly responded to. "Shakespeare says, 'silence is the perfect sign of appreciation;' I were but little thankful if I could say how much. I cannot do better than extend this quotation to those so kind to me."

The December meeting was held on the 8th instant, with the following members of the profession present: Drs. T. S. Allen, H. P. Eves, Charles T. Goentner, J. R. Hart, W. H. Hoskins, W. S. Kooker, J. T. McAnulty, L. Pearson, W. L. Rhoads, and a few students.

A report of the Committee on Certificates was made, the certificates issued to those present, and the committee, having completed its task, was discharged.

Under unfinished business, the election of Dr. E. A. A. Grange as an honorary member was in order. His election to honorary membership was unanimous.

The following amendment to Article II., Section 3, of the By-laws was offered by Drs. Leonard Pearson and W. H. Hoskins: "Candidates for honorary membership must be proposed in writing by two active members of the Association and their names referred to the Board of Trustees, and acted upon at a following meeting." The applications of Dr. Thomas B. Rayner, of Chestnut Hill, and H. A. Christman, of the same place, having been favorably reported by the Board of Trustees, were acted upon by the Association and unanimously elected to active membership.

Dr. Ravenal, of the State Bacteriological Laboratory, now gave an interesting talk on "Bacteriology in its Relation to Veterinary Science," during which he spoke of the great interest taken in science by a great number of veterinarians, the works of some having been epoch markers—the field of preventive medicine having so far proven more productive to the veterinarian than to his brothers in the healing art. Based on bacteriology we have preventive inoculations. He spoke of the three distinct types of vaccination, quarantine, etc. He then spoke of the best way for the general practitioner to assist the specialist in bacteriology in his work for their mutual benefit. He said the theory of burying anthrax cases seven or eight feet deep to prevent further infection, while practically safe, was not literally so, as experiments have shown that the changes due to decomposition develop heat enough to allow the formation of spores which might be brought to the surface by earth-worms. He spoke of osteoporosis and meningitis as being of peculiar interest alike to the practitioner and the scientist.

Dr. Hoskins asked by what means they might become certain as to the diagnosis of what we at times know as dumb rabies. Dr. Goentner, having seen a number of these cases, said he thought in the majority of cases it was but a form of meningitis. Dr. Ravenal now explained how the diagnosis might be verified by successive inoculations, the germ constantly becoming more virulent. Dr. Pearson spoke of a culture being carried through eight generations in the rabbit.

Dr. Hoskins now spoke of a committee from the Milkmen's Association having waited upon him and asking that they be granted a hearing on the milk question, as they felt Dr. Morris had been heard in opposition to them. It was moved and decided to invite Mr. George Abbott to give a half-hour talk at the January meeting on the milk question. W. L. Rhoads,

Secretary.

